# Single-Cell Analysis of Growth and Cell Division of the Anaerobe *Desulfovibrio vulgaris* Hildenborough

**DOI:** 10.3389/fmicb.2015.01378

**Published:** 2015-12-08

**Authors:** Anouchka Fievet, Adrien Ducret, Tâm Mignot, Odile Valette, Lydia Robert, Romain Pardoux, Alain R. Dolla, Corinne Aubert

**Affiliations:** ^1^Centre National de la Recherche Scientifique, Laboratoire de Chimie Bactérienne UMR 7283, Aix Marseille UniversitéMarseille, France; ^2^INRA, UMR1319 MicalisJouy-en-Josas, France; ^3^AgroParisTech, UMR MicalisJouy-en-Josas, France

**Keywords:** sulfate-reducing bacteria, *Desulfovibrio*, anaerobic bacteria, cell division, oxygen stress adaptation

## Abstract

Recent years have seen significant progress in understanding basic bacterial cell cycle properties such as cell growth and cell division. While characterization and regulation of bacterial cell cycle is quite well-documented in the case of fast growing aerobic model organisms, no data has been so far reported for anaerobic bacteria. This lack of information in anaerobic microorganisms can mainly be explained by the absence of molecular and cellular tools such as single cell microscopy and fluorescent probes usable for anaerobes and essential to study cellular events and/or subcellular localization of the actors involved in cell cycle. In this study, single-cell microscopy has been adapted to study for the first time, in real time, the cell cycle of a bacterial anaerobe, *Desulfovibrio vulgaris* Hildenborough (DvH). This single-cell analysis provides mechanistic insights into the cell division cycle of DvH, which seems to be governed by the recently discussed so-called incremental model that generates remarkably homogeneous cell sizes. Furthermore, cell division was reversibly blocked during oxygen exposure. This may constitute a strategy for anaerobic cells to cope with transient exposure to oxygen that they may encounter in their natural environment, thereby contributing to their aerotolerance. This study lays the foundation for the first molecular, single-cell assay that will address factors that cannot otherwise be resolved in bulk assays and that will allow visualization of a wide range of molecular mechanisms within living anaerobic cells.

## Introduction

Sulfate-Reducing Microorganisms (SRM) constitue a phylogenetically diverse group of anaerobe bacteria and archaea that, occupy important environmental niches and have potential for significant biotechnological impact. SRM gain energy for biosynthesis and growth by coupling the oxidation of organic compounds or molecular hydrogen to the reduction of sulfate into sulfide (Thauer et al., 2007). Sulfate reduction can account for more than 30% of the organic carbon mineralization in marine sediments (Jørgensen, 1982), placing SRM as a keystone of both the sulfur and carbon geochemical cycles. Despite the anaerobic nature of this metabolic process, sulfate-reducing activity is not confined to permanently anoxic habitats. The view that SRM are unable to cope with oxygen began to change in the 1990s when sulfate reduction was observed in oxic biotopes (Canfield and Des Marais, 1991). During the last decades, SRM have been not only observed in oxic zones of numerous biotopes, including marine and fresh water sediments, but also have appeared to be more abundant and more active than the SRM observed in the neighboring anoxic zones, presumably due to enhanced access to bio-degradable organic matter (Sass et al., 1998; Ravenschlag et al., 2000; Mussmann et al., 2005).

SRM could play both favorable and unfavorable roles: although they are involved in the bioremediation of aromatic and chlorinated hydrocarbons and toxic metals such as U(VI) and Cr(VI) in contaminated soils, SRM are also involved in biocorrosion of petroleum pumping equipment, storage tanks, and pipelines (Boothman et al., 2006) as well as in the production of hydrogen sulfide, a strong neurotoxic (Zhou et al., 2011). Additionally, SRM are part of the normal human intestinal flora (Jia et al., 2012).

For all these reasons, the past 10 years were spent to understand the biochemistry, molecular biology, physiology, and ecology of SRMs using systems biology approaches such as genetics, transcriptomics, proteomics, metabolomics, and metagenomics (Zhou et al., 2011).

While all these approaches have been used to study SRM at the population scale, to our knowledge, no studies of SRM have been performed at the single-cell level. Then mechanisms like growth and cell division are poorly documented for SRM and, to a larger extent, for strictly anaerobic microorganisms. On the contrary, cell division mechanism in aerobes is largely documented, mainly because of the development of single-cell microscopy and fluorescent probes, which have been extensively used to describe cellular events and/or the subcellular localization of the major actors involved in cell cycle.

Dividing cells have to coordinate DNA replication, chromosome segregation and cytokinesis. In bacteria, cell cycle progression is generally coupled with cellular growth. Cellular growth and cell division have been shown to be spatially and temporally regulated but the molecular basis of this regulation is still unclear and seems to depend on the organism studied. For example, although FtsZ is the most highly conserved cell division protein throughout aerobic and anaerobic bacteria (Rothfield et al., 2005; Harry et al., 2006; Thanbichler and Shapiro, 2006; Barák and Wilkinson, 2007; Wu and Errington, 2012), the mechanisms positioning the FtsZ ring at the mid-cell site is governed by several different inhibitors such as Min and NO systems in aerobic rod shaped bacteria *E. coli* and *B. subtilis* (Bi and Lutkenhaus, 1991; de Boer et al., 1992; Bernhardt and de Boer, 2005; Wu and Errington, 2012) and by other alternative regulators such as ParA in *Corynebacteria* (Donovan et al., 2013), MipZ in *Caulobacter crescentus* (Thanbichler and Shapiro, 2006), or pomZ in *Myxoccus xanthus* (Treuner-Lange et al., 2013).

Coordination of cell growth and division is essential to maintain the cell size in bacteria but still remains largely mysterious. Historically, cell size homeostasis has been described in terms of two models of control, “timer” and “sizer” (Turner et al., 2012): the latter, in which the cell actively monitors its size and triggers the cell cycle once it reaches a critical size, and the former, in which the cell attempts to grow for a specific amount of time before division. However, very recently, the ability to analyze bacteria at the single-cell level in real time provided new and important insights into the cell-size maintenance mechanism and revealed a new strategy, called the incremental model, which is based on a constant size increment between two successive events of the cell cycle (Campos et al., 2014; Soifer et al., 2014; Taheri-Araghi et al., 2015).

All these new results onto the progression of cell cycle of aerobic microorganisms have been obtained thanks to the development of single-cell experiments in real time. However, while imaging living cells in aerobic conditions has become a standard procedure, performing live-cell imaging under anaerobic conditions is a major technical challenge and might explain the lack of information on bacterial cell processes such as growth and progression of the cell cycle in anaerobe microorganisms.

Here, specific microscopy chambers were designed to monitor, in live cells, the cell cycle of *Desulfovibrio vulgaris* Hildenboroug (DvH), an anaerobic sulfate-reducing bacterium, and determine the pedigrees of growing DvH cells in anoxic conditions. In addition, these chambers allowed the observation at the single-cell level of the response of DvH to oxygen. We first performed time lapse microscopy experiments to monitor the growth and division of single cells within microcolonies in anaerobic conditions. Our results show that cell size control in DvH is well-described by the incremental model, showing for the first time that the proposed incremental model can be applied to some anaerobic microorganisms. We then studied the response of DvH cells to a transient oxygen exposure and found that cell division was reversibly blocked in the presence of oxygen. We propose that it constituted a strategy for anaerobic cells to cope with transient exposure to oxygen that they may be encountered in their natural environment.

## Materials and methods

### Bacterial strains, plasmids, primers, and growth conditions

All strains and plasmids used in this study are listed in Table S1. The primers used in this study are listed in Table S2. *Escherichia coli* DH5α, WM3064, and EC448 were grown at 37°C in Luria-Bertani medium supplemented with the appropriate antibiotic when required (0.15 mM chloramphenicol and 0.27 mM ampicillin). *E. coli* WM3064 was grown in the presence of 0.3 mM 2,6-diaminopimelic acid (DAP). Cultures of DvH were performed in either C medium (Postgate et al., 1984) or LS4D-YE medium at 33°C in an anaerobic chamber (COY Laboratory Products) filled with a 10% H_2_-90% N_2_ mixed-gas atmosphere. One liter of LS4D-YE medium (pH 7.2) contains 50 mM NaSO_4_, 60 mM sodium lactate, 8 mM MgCl_2_, 20 mM NH_4_Cl, 2.2 mM K_2_PO_4_, 0.6 mM CaCl_2_, 30 mM piperazine-*N*,*N*′-bis(ethanesulfonic acid) (PIPES) buffer, 1 g/L of yeast extract, 10 mM NaOH, 1 ml of Thauers vitamins and 12.5 ml of trace minerals. The final mixture was autoclaved and used. The medium was supplemented with 0.17 mM kanamycin and 0.15 mM thiamphenicol when mentioned.

### DNA manipulations

Standard protocols were used for cloning and transformations. All restriction endonucleases and DNA modification enzymes were purchased from New Englands Biolabs. Polymerase chain reactions (PCRs) were performed with PrimeSTAR™HS DNA Polymerase from Takara. DNA ligations were performed with LigaFast™ Rapid DNA Ligation System from Promega. The High Pure Plasmid Isolation kit from Roche was used to purify plasmidic DNA. Chromosomal DNA was purified using the Wizard Genomic DNA purification kit from Promega. DNA fragments and plasmids were excised or purified using the MinElute kits from Qiagen.

### Construction of the DvH (pBMC6 *pC*_3_*::gfp*) strain

In order to express the GFP in DvH, a transcriptional fusion between the constitutive promoter pC_3_ of the gene *cyc* encoding for c_3_ cytochrome (Mr13000) and the *gfp* gene was constructed. To construct the strain DvH (pBMC6*pC*_3_*::gfp)*, the pC_3_ promoter was amplified from the DvH genomic DNA by using the primer couple Promcyc_*Hind*III/Promcyc_*Sal*I_*Nde*I. The amplicon was cut with *Hind*III and *Sal*I and cloned into pBMC6 cut with the same enzymes to give the plasmid pBMC6*pC*_3_. The *gfp* gene was then amplified from pEGFP-N1 plasmid by using the primers NterGFP-*Nde*I and CterGFP-*Sac*I. The amplicon was then cut with *Nde*I and *Sac*I and subcloned into the pBMC6 *pC*_3_ plasmid cut with the same enzymes. The obtained plasmid was transferred into DvH by electroporation as described by Fiévet et al. (2011). The presence of the pBMC6 *pC*_3_*::gpf* plasmid in DvH cells was controlled by PCR by using the primer couple promcyc_*Hind*III/CterGFP-*Sac*I.

### Construction of the DvH *(ftsZ-gfp)* strain

A non-replicative plasmid with the fusion *ftsZ-gfp* was constructed, allowing chromosomic insertion of the plasmid into the *ftsZ* locus. This integration allowed replacement of the endogenous *ftsZ* gene by the *ftsZ-gfp* fusion and gave a *ftsZ* gene copy devoided of promoter (Figure S1). The *ftsZ* gene was amplified from the DvH genomic DNA by using the primer couple (NterFtsZ-*Xho*I/ CterFtsZlink-*Nde*I-*Spe*I) with the addition of a linker, coding for four arginine in the C-terminal region of FtsZ. The obtained amplicon was cut with *Xho*I and *Spe*I and then cloned into the plasmid pNot19Cm-Mob-XS cut with the same enzymes to form the pNot19Cm-Mob-XS-*ftsZ* plasmid. The primer CterFtsZ-*Nde*I-*Spe*I allowed the insertion of an *Nde*I site upstream the *Spe*I site. After amplification of the *gfp* gene by PCR from pEGFP-N1 plasmid by using the primer couple NterGFP-*NdeI*/CterGFP-*SpeI, gfp* was subcloned into *Nde*I and *Spe*I sites in the pNot19Cm-Mob-XS-*ftsZ*. The obtained plasmid, pNot19Cm-Mob-XS-*ftsZ-gfp*, was then transferred into *E. coli* MW3064 and subsequently transferred by conjugational gene transfer into DvH. Cells carrying the chromosomal recombination with the target fusion were selected for their resistance to thiamphenicol and checked by PCR using primers couple: FtsA_dir/CterGFP-SpeI.

### Western blotting experiments

Production of the soluble GFP and the FtsZ-GFP fusion in DvH (pBMC6*pC*_3_*::gfp*) and DvH (*ftsZ*-GFP), respectively, was analyzed by western blot using an anti-GFP. Cells were grown in medium C until the OD_600_ reached 0.4–0.6. To prepared samples, 0.5 OD_600_ units of DvH cultures were centrifuged and the pellet was resuspended in 50 μL of 2X loading buffer (120 mM of Tris-HCl pH 6.8, 20% of glycerol and 0.2% of bromophenol blue) supplemented with 0.69 mM of SDS and 10 mM of DTT. Then, samples were boiled for 10 min. After separation by electrophoresis in 12.5% SDS polyacrylamide gel, proteins were transferred onto a nitrocellulose membrane followed by blocking of the membrane with PBS containing 3% BSA and 0.5% Tween-20 for 1 h at room temperature. After three washes in PBS buffer containing 0.1% of Tween-20, the membrane was incubated overnight with a polyclonal home-made rabbit anti-GFP serum diluted at 1:10^8^. The membrane was then washed three times in PBS buffer supplemented with 0.1% of Tween-20 and incubated 1 h at room temperature with a HRP-conjugated anti-rabbit secondary antibody from Thermo Scientific (1:5000 in PBS 3% BSA, 0.5% Tween-20). After two washes in PBS buffer supplemented with 0.1% of Tween-20 followed by two washes in PBS buffer, SuperSignal® West Pico Chemiluminescent Substrate kit (Thermo Scientific) was used for detection according to the manufacturers' instructions. The signal detection was realized by using ImageQuant LAS 4000 mini from GE Healthcare.

### The anaerobic and controlled atmosphere observation chambers

The anaerobic observation chamber is adapted from the microfluidic system described by Ducret et al. (2009). Briefly, the cells are confined between a coverslip placed on an adapter and a transparent lid (Figure S2A). The transparent lid is made from Poly(methyl methacrylate; PMMA; Figure S2B, Table S3). On the inner side of the lid, a groove designed to receive a 1.6 mm diameter O-ring made from elastomer (121.9 mm of circumference) ensures air-tightness. The adapter is made from Aluminum Alloys AU 4 G and is designed to match with No. 1.5 coverslips (24 × 50 mm; Figure S2D, Table S3). To keep the assembly on the adapter and ensure air-tightness, six fastening screws are disposed at equal distance around the coverslip. This arrangement ensures proper load distribution during clamping. For the controlled-atmosphere version, the transparent lid is drilled out to receive tubing connectors (GE18-1003-68 Sigma, France) with 0.5 mm diameter O-ring made from elastomer (8.16 mm of circumference) to ensure air-tightness. Tubing connectors are tightened through the transparent lid using custom hollowed brass screws (Figure S2C, Table S3).

### Time-lapse microscopy

**Figure 1:**
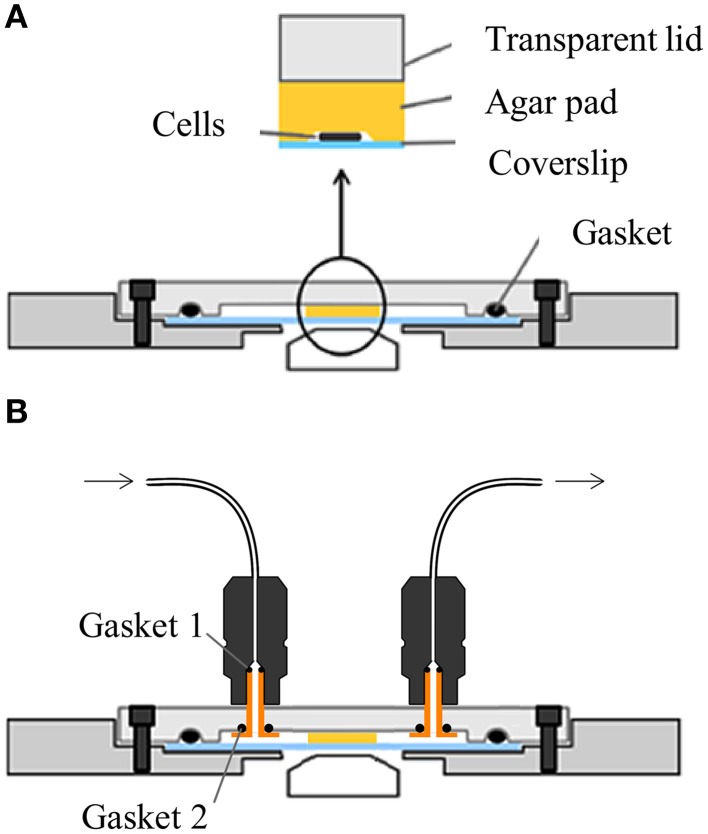
**(A)** Layout of the anaerobic observation chamber: Cells were placed between a coverslip and an agar pad (a thin layer of solid medium). The two parts of the system are sealed together. The use of a vacuum grease coated gasket improved the sealing of the chamber. **(B)** Layout of the controlled-atmosphere chamber. The lid of the chamber is pierced of an entrance and an exit allowing the injection and evacuation of gas. The gasket couple one precludes air entrance between the lid and the injection system. The gasket couple two prevents air entrance near tubing inserted in the injection system.

DvH cells were grown in LS4D-YE medium at 33°C until an OD_600_ of approximately 0.3–0.4. One microliter of cell culture was placed between the coverslip and a thin layer of LS4D-YE medium supplemented with 1.5% of Phytagel™ from Sigma-Aldrich (Figure 1A) that was previously prepared under anaerobic conditions. The anaerobic chamber was hermetically closed in the absence of oxygen using a lid with a gasket coated with vacuum grease. Once sealed, the observation chamber was transferred to a standard temperature-controlled inverted microscope, without introducing oxygen. For experiments with controlled-atmosphere, a specific lid equipped with two luer systems to allow flow injection was used (Figure 1B). This system allowed the injection of a defined gas by connecting it to an injector pump linked to a Pegas 4000 MF Gas Mixer (Columbus instruments). To apply a constant stress of 0.05% oxygen to the cells, the gas mixer was regulated to inject 400 mL/min of nitrogen and 1 mL/min of 20% O_2_-80% N_2_ gas mix. For switching experiments, the injector pump was connected to a solenoid that was connected to the 0.05% O_2_ and N_2_ entries to restore aerobic conditions. Time-lapse experiments were performed with a TE2000-E-PFS inverted epifluorescence microscope (Nikon, France) at 33°C. Images were recorded with a CoolSNAP HQ2 (Roper Scientific, Roper Scientific SARL, France) and a 100x/1.4 DLL objective. Image processing was controlled by an automation script under Metamorph 7.5 (Molecular Devices, Molecular Devices France, France), which was previously developed in the laboratory. Phase-contrast and fluorescence images, when required, were acquired every 5 and 20 min, respectively. Final image preparation was performed using ImageJ (Schneider et al., 2012). All cell-length and division-time measurements were determined manually. All these measurements have been used to determine the cell elongation rate by using the following equation: elongation rate = (ln(cell-length at division)-ln(cell-length at birth))/division time.

### Fluorescent, single time-point experiments

DvH (f*tsZ*-GFP) cells were grown until the middle of the exponential growth phase (OD_600nm_ of approximately 0.4 to 0.5). Cultures (200 μL) were centrifuged, and the pellet was resuspended in 100 μl of 10 mM Tris-HCl (pH 7.6), 8 mM MgSO_4_, and 1 mM KH_2_PO_4_ buffer (TPM buffer) containing 5 ng/μL of 4′,6-diamidino-2-phenylindole (DAPI). After 20 min of incubation in the dark, the cells were washed three times in TPM buffer. The DNA was stained under anaerobic conditions to limit the exposure of the cells to air. The pictures were acquired after 10 min of air exposure, which was required for oxygen GFP maturation. The cells were placed between a coverslip and an agar pad of 2% agarose supplemented with 10 ng/μL FM4-64® from Invitrogen. Pictures were acquired with a Nikon TiE-PFS inverted epifluorescence microscope, 100x NA1.3 oil PhC objective (Nikon), and Hamamatsu Orca-R2 camera. For fluorescent images, a Nikon intenslight C-HGFI fluorescence lamp was used. Specific filters were used for each wavelength (Semrok HQ DAPI/CFP/GFP/YFP/TxRed). Image processing was controlled by the NIS-Element software (Nikon).

## Results

### Design of an anaerobic chamber device

Following *in vivo* and in real time the complex work of the cellular machinery is a powerful approach for evaluating the dynamics of bacterial processes such as growth and cell division. Unfortunately, the sensitivity of anaerobic organisms to oxygen makes the observation of their cell cycle extremely difficult. Until now, most commercial or pre-existing observation chambers have not been suitable for anaerobic microorganisms because they are not fully resistant to oxygen (Ducret et al., 2009; Charvin et al., 2010). *In vivo* microscopic analysis of these organisms was therefore limited by the lack of observation chambers hermetic to the outside air and wherein the atmosphere could be accurately controlled. The studies of physiology and adaptive responses of anaerobic microorganisms therefore needed to lift this technical obstacle by developing a hermetic observation chamber adaptable on a standard microscope and in which atmosphere can be controlled and modified. We thus set up a specific observation chamber, adapted from the microfluidic system described by Ducret et al. (2009), that held the principle of observation systems conventionally used in which cells were confined between a coverslip and a thin layer of agar with a custom hermetic chamber (Figure 1A). Schematically, the cells were placed on a coverslip and overlaid with a 0.5 mm thin layer of solid medium culture with Phytagel™in an anaerobic glove box and left to dry gently to absorb the cells onto the agar substrate. The microscopic chamber was then sealed with a transparent lid containing a gasket allowing the air tightness of this chamber (Figure 1A). For observation, the chamber was then put onto a Nikon epifluorescence microscope located outside the anaerobic chamber.

To check that our new chamber device was airtight, it was filled up under anaerobic conditions with a DvH strain producing soluble GFP, DvH (pBMC6*pC*_3_*::gfp*), and then incubated overnight outside the anaerobic chamber. The growth of DvH in the chamber and the absence of fluorescence under these conditions showed that this system was hermetic to outside air (data not shown). As control when oxygen was introduced into the observation chamber, a fluorescent signal was detected after 5 min. As GFP needs to be in contact with a minimum concentration of 10^−5^% of O_2_ to fluoresce (Hansen et al., 2001), the absence of fluorescent signal in DvH (pBMC6 *pC*_3_*::gpf*) cells cultured in the observation chamber demonstrated that no more than 10^−5^% of O_2_ penetrated into the observation chamber. Our hermetic observation chamber can be thus used to study in real time the growth at the single-cell level of anaerobic microorganisms like DvH.

### The cell cycle of DvH, as revealed by microscopic single-cell analysis

**Figure 2:**
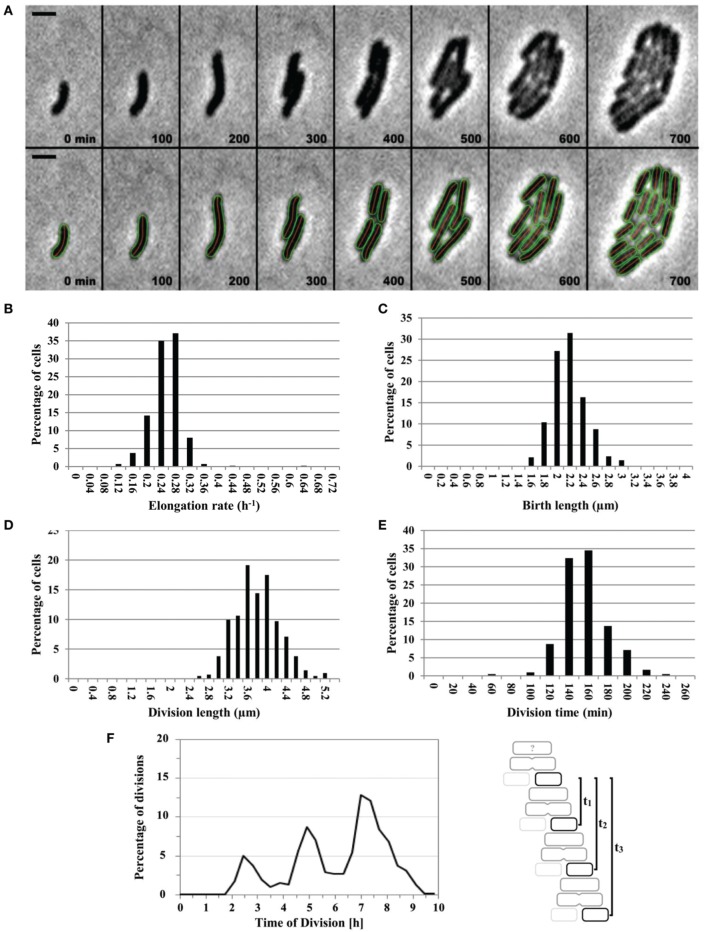
**The cell cycle of DvH is temporally and spatially regulated**. **(A)** Sequence of images showing several rounds of division from a single DvH WT cell in the absence of oxygen. Cell contours retrieved by the annotation software are added for clarity. Time points are indicated for each time frame. (Scale bar = 2 μm). **(B–E)** Distribution of the elongation rate **(B)**, birth length **(C)**, division length **(D)**, and division time **(E)** as percentage of dividing DvH cells. **(F)** The percentage of cell division events as a function of time (*n* = 423). As the initial state of individual cell at the beginning of the experiment was unknown, the time of each consecutive division (*n* = 423) is computed from the time of the first division event for each lineage (see diagram). The two first divisions were removed for the calculation of the division time of each lineage.

The hermetic observation chamber described above was used to monitor for the first time a complete division cell cycle of DvH (Figure 2A). To follow the dynamics of DvH cell growth, the length vs. time of individual DvH cells growing in LS4D YE medium was measured from birth to division. The limited accuracy of cell size measurements did not allow an accurate characterization of the growth law. Nevertheless, our results were compatible with the exponential elongation observed in many other bacteria (Campos et al., 2014). Statistical analyses of DvH single-cell cycle were performed to determine DvH cell division parameters. For that, birth length and cell size at division were manually measured and time of division as well as elongation rate were determined. Under anaerobic conditions, DvH grew with an average doubling time of 2.48 ± 0.39 h (Figures 2A,E), which, surprisingly, was about twice as fast as the doubling time observed in liquid culture (≈5.4 ± 0.72 h). Single-cell analysis suggested that the elongation rate followed a normal distribution described by an average value of 0.23 ± 0.04 h^−1^ (Figure 2B). Most interestingly, the birth length and the length at division followed a narrow distribution with a mean of approximately 2 and 3.7 μm and with a CV of 0.13 and 0.12 respectively, suggesting the existence of mechanisms controlling cell size (Figures 2C,D). Lastly, by tracking the timing of cell division events over generations for each lineage, the occurrence of division was observed at regular intervals (Figure 2F) suggesting that the timing of birth events was also remarkably conserved over divisions. Together, these data strongly suggested that the cell cycle of DvH in the absence of oxygen was tightly controlled in time and place resulting in the production of a remarkably homogenous cell size population.

### Mechanism responsible for the cell size maintenance in DvH

**Figure 3:**
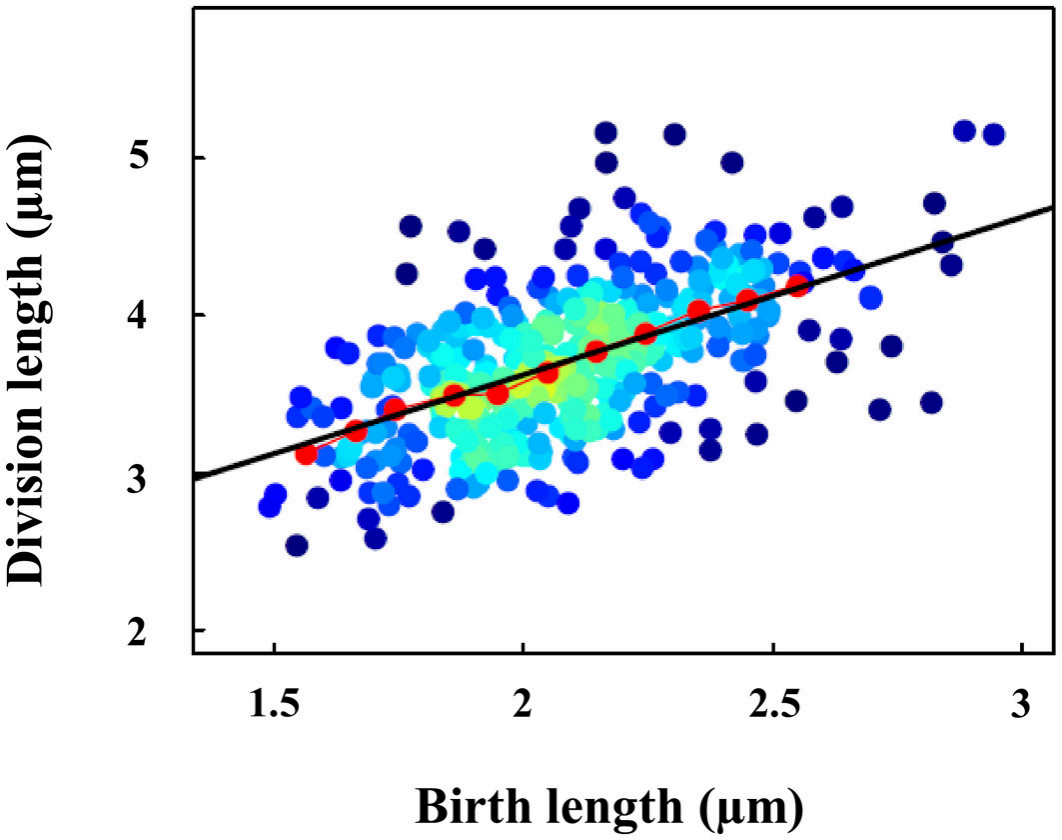
**Positive correlations between size at birth and size at division in DvH daughter cells**. The color of the dots (blue to yellow) represents the local density. Red dots show data binned according to the size at birth. The prediction with the incremental model is indicated with a black line (slope = 1).

The mechanism responsible for cell size homeostasis remains, to date, undetermined. However, the nature of cell-size maintenance can be revealed by analyzing the relationship between the birth length and length at division for each individual cell (Figure 3).

As shown in Figure 3, birth length and cell length at division are positively correlated. This correlation suggested that size control did not follow the classical model of “sizer” in which division occurred at a critical size. Figure 3 shows that experimental data binning according to the size of birth (red dots) fitted with the prediction of the incremental model (black line). It thus suggested that, DvH cell division followed the incremental model, in which cells added a constant volume at each generation irrespective of the birth size to maintain size homeostasis (Campos et al., 2014; Jun and Taheri-Araghi, 2015; Taheri-Araghi et al., 2015). The absence of correlation between elongation length and cell length at birth strengthens the incremental model hypothesis (Figure S3).

### Septum formation in DvH

**Figure 4:**
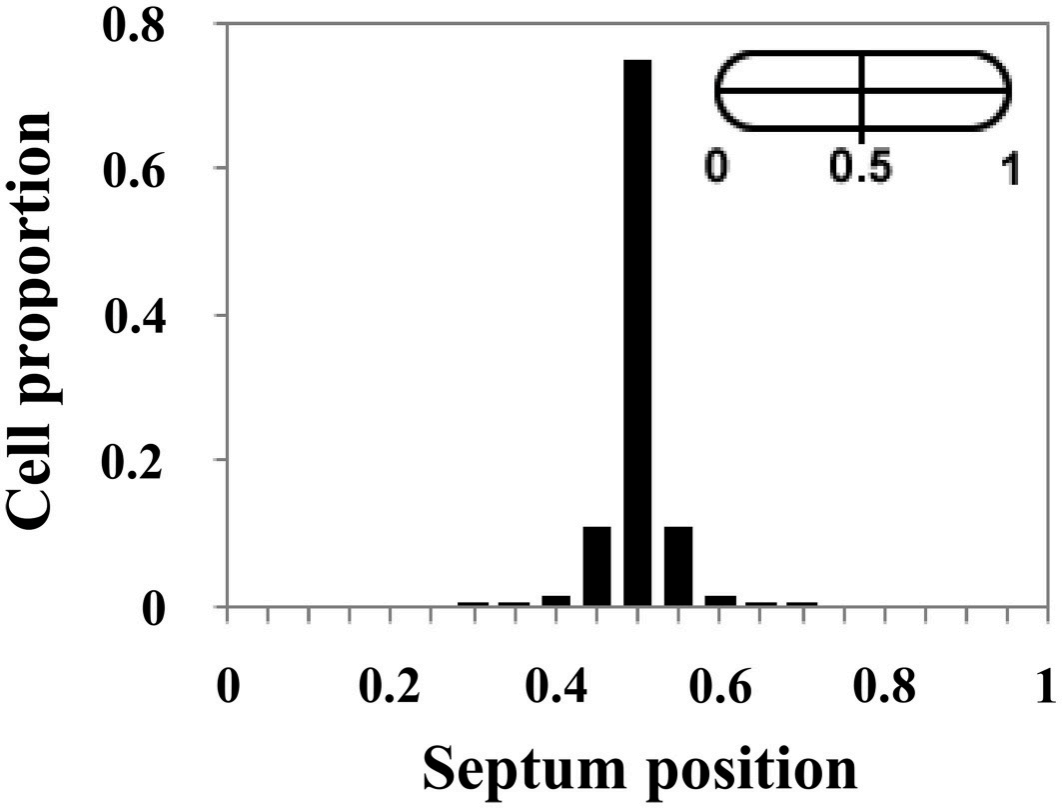
**Division takes place in the middle of DvH cells**. Distribution of the division site placement in WT cells (*n* = 440). The midcell position corresponds to 0.5.

Cell size control can act through the limitation of variations of cell size at division as well as through the precise positioning of the division site at mid-cell. To determine the division symmetry in DvH, the distance between the division septum and the nearest cell pole was measured in single cells. As observed in Figure 4, 97% of cells exhibited a division site located between 40 and 60% of the cell size. The division site is located at the middle of the cells with a mean of 46.8 ± 2.35% with a CV of 0.05.

To assess information on the septum formation in DvH, a fluorescent protein fusion of FtsZ was constructed by fusing GFP to the C-terminus of FtsZ, to replace the endogenous *ftsZ* gene by the *ftsZ-GFP* fusion gene in the DvH chromosome (Figure S1). Cells expressing the FtsZ-GFP fusion had the same growth characteristics, mid-cell placement of the division site and timing of division as the wild-type cells, suggesting that the GFP-fusion was fully functional (Figure S4). Additionally, western blot analysis using anti-GFP antibodies revealed only one band corresponding to the fusion protein (Figure S5) and suggested that the integrity of the fusion protein was conserved. Without an anti-FtsZ antibody usable on DvH extract due to a lot of non-specific signals on the western blot, the absence of production of the native FtsZ cannot be excluded but as transcription of the endogenous *ftsZ* gene devoided of its own promoter represented only 1% of the regular transcription rate as quantified by qRT-PCR (data not shown), one could consider that the production of native FtsZ, if it existed, would be weak.

**Figure 5:**
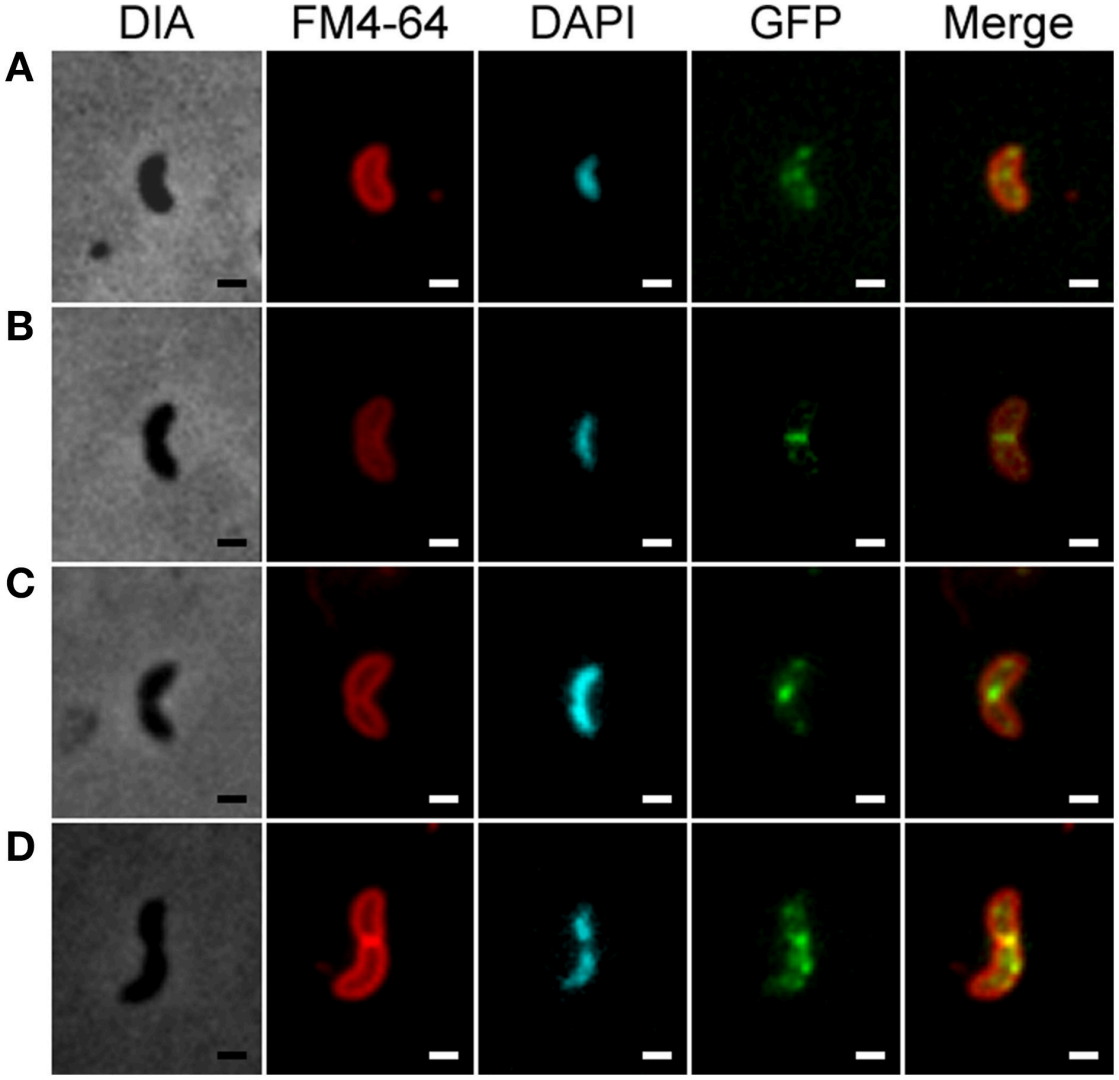
**The Z-ring presents two types of localization in DvH**. Simultaneous localization of FtsZ-GFP, the cell membrane (FM4-64) and the nucleoid (DAPI) during consecutive and representative steps of the cell cycle of DvH: initiation **(A)**, elongation **(B)**, septation **(C)** and division **(D)**. In each case, the first column represents the phase-contrast images (DIA), the second represents the membrane localization (FM4-64), the third represents the nucleoid localization (DAPI), the fourth represents the FtsZ-GFP localization, and the fifth represents an overlay of FtsZ-GFP and FM4-64 fluorescent signals. Scale bar = 1 μm.

Because GFP did not fluoresce in the absence of oxygen (Hehl et al., 2000), cells were first grown under anaerobic conditions, and then briefly exposed to air to allow the GFP to fluoresce. In order to visualize the effect of oxygen on the integrity and location of proteins, we observed, by fluorescent microscopy, the positioning of the FtsZ-GFP protein fusion at different exposure times to air (Figure S6). Delocalization of the FtsZ ring was observed after later than 1 h of oxygen exposure (Figure S6). The pictures were acquired less than 10 min after contact with air, a time of incubation that was not sufficient to significantly affect the localization of the FtsZ-GFP fusion (Figure S6). To follow the progression of the cell cycle, nucleoids and cell membranes were also stained with DAPI and FM4-64, respectively. The localization of FtsZ-GFP during the cell cycle is shown in Figure 5. Before cell elongation and chromosome segregation, FtsZ presented two types of localization: (i) a series of bright dots that were localized on the periphery of the cells, connected by fluorescent lines (Figure 5A) and/or (ii) a clear Z ring at mid-cell (Figure 5B). As observed, the bright dots were associated with a diffuse DNA content that became compact as the Z ring developed (Thanedar and Margolin, 2004; Peters et al., 2007). Once the chromosome was segregated and the septum was initiated, only the Z ring was observed at mid-cell which disappeared when cell division was completed (Figures 5C,D). It should be noted that in the early stage of the cell cycle, when the Z-ring was formed, the Z-ring seemed to overlap with the DNA (Figure 5B). Together, these data suggest that DvH displays dynamic localization of FtsZ, which was comparable to model organisms such as *E. coli* (Harry et al., 2006).

### Effect of low-oxygen exposure on DvH division

A lot of studies have been done to understand how DvH responds to oxidative stress in bulk assays (Dolla et al., 2006). However, none of them, showed the effect of oxygen on DvH cell division at the single-cell level. For this purpose, another hermetic microscopic chamber that is able to rapidly switch the composition of the atmosphere was developed (Figure 1B).

**Figure 6:**
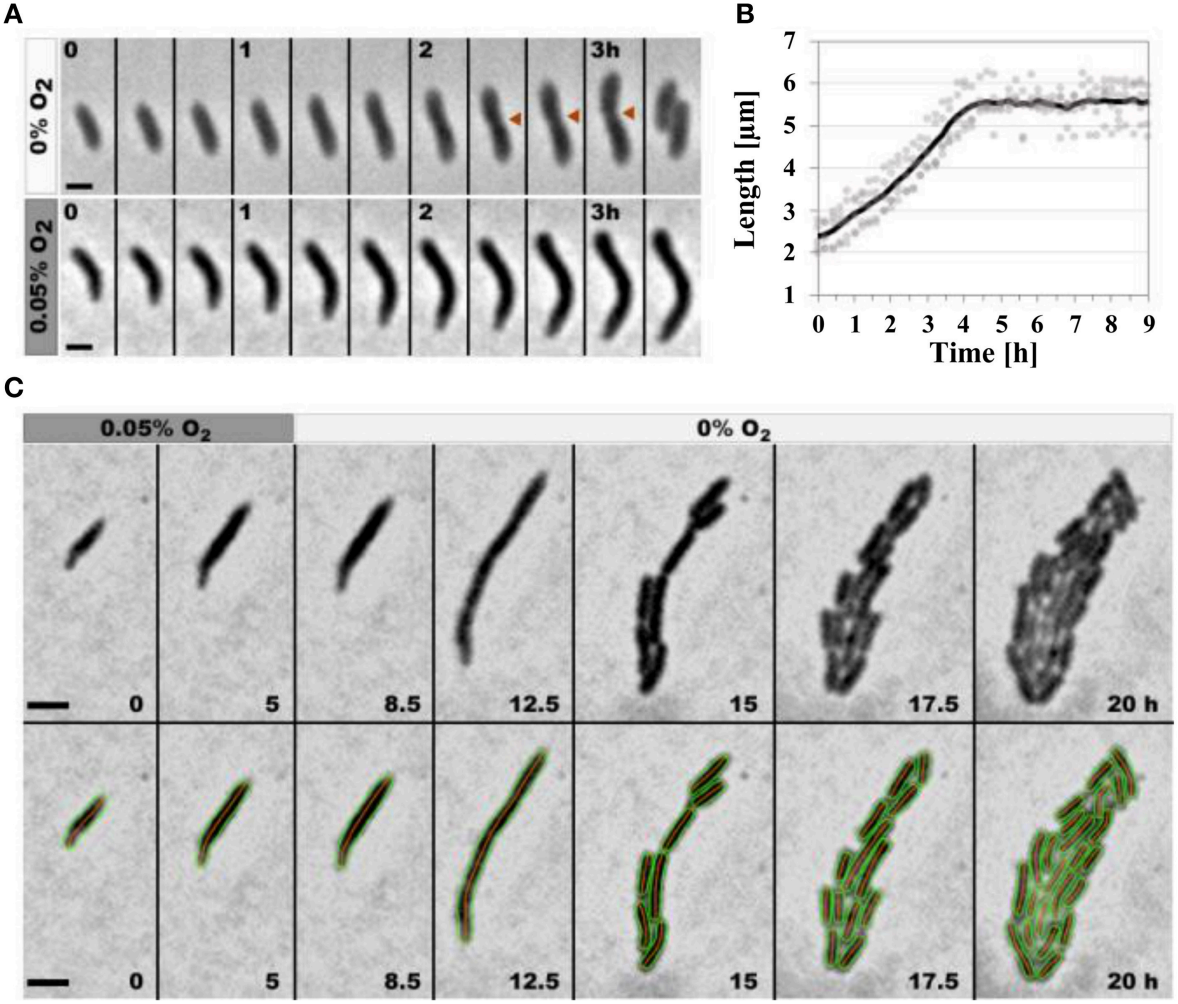
**Low-oxygen exposure leads to reversible filamentation in DvH**. **(A)** Sequence of images showing the growth of a single DvH cell in the absence (top) or presence of oxygen (0.05%; bottom). Red arrows highlight the constriction site preceding cell division. **(B)** Quantification of the cell length with time (h) when cells were grown in the presence of oxygen (0.05%). The dark line indicates the median values (*n* = 20). **(C)** Sequence of images showing several rounds of division from a single DvH cell first in the presence of 0.05% oxygen for 5 h, followed by a period of 15 h under anaerobic conditions. Cell contours retrieved by the annotation software are added for clarity. Time points are indicated for each time frame (Scale bar = 2 μm).

To test the response of DvH cells at the single-cell level, cells were first grown under anaerobic conditions, then placed in this controlled-atmosphere chamber and finally exposed to different concentrations of oxygen. Cells cultured in atmosphere containing up to 0.02% oxygen were not affected and presented the same growth parameters as cells observed in the absence of oxygen (data not shown). When oxygen concentration was higher than 0.05%, cells stopped growing suggesting that both division and elongation were affected (Figure 6A). In between these two oxygen concentrations (0.02 and 0.05%), cell division was affected and filamentation was observed in 95% of the cells: cells elongated at the same rate as those observed in the absence of oxygen but did not septate, suggesting that cell division was prevented (Figure 6B).

To test the reversibility of oxygen exposure, cell growth was monitored in the presence of oxygen (0.05%) for 5 h and then in the absence of oxygen (anaerobic condition) for additional period of 15 h (Figure 6C). As expected, exposure to 0.05% oxygen led first to filamentation and then to a complete arrest of growth. However, 3 h after the transition from aerobic to anaerobic conditions, cells resumed elongation but did not septate, leading to the formation of long filamentous cells (longer than 10 μm). Seven hours after the transition, cell division was resumed, and cells progressively regained the typical size and morphology of cells growing in anaerobic conditions. Together, this suggests that the cell division of DvH is transiently and reversibly blocked during oxygen exposure (Figure 6C).

## Discussion

During the last decades, the characterization of cell division in aerobic microorganisms has been greatly improved by the advent of single-cell approaches, while the characterization of the cell division in anaerobic microorganisms has remained mostly unexplored. Most existing observation chambers were not suitable for the establishment of anoxic conditions, precluding any single-cell analysis of anaerobic microorganisms. In this context, we have developed an oxygen-tight observation chamber to observe the cell cycle of an anaerobic sulfate reducing bacterium, *D. vulgaris* Hildenborough. To our knowledge, this is the first observation of the cell cycle of an anaerobic organism at the single-cell level. This observation chamber provides a new approach to image anaerobic microorganisms at the single-cell level and permits real time microscopy. Therefore, this technique is suitable for a wide range of other obligate anaerobic, aerotolerant, or microaerophilic microorganisms. This device is an asset of choice for the study of cellular processes or molecular mechanisms developed by anaerobes hitherto unexplored (such as cell division, motility, or chemotaxis).

Single-cell analysis of growing cells suggests that the cell cycle of DvH is spatially and temporally regulated in the absence of oxygen. Birth and division lengths exhibit relatively narrow distributions, suggesting the existence of size control mechanisms. Moreover, the correlation between the cell size at birth and the cell size at division suggests that the cells do not divide at a critical size. Two different models could explain such a correlation. If cellular growth is linear, cell size control could rely on a timer mechanism. Nevertheless, although linear growth cannot be excluded based on our data, most bacteria are known to grow exponentially (Campos et al., 2014). In this case, the relation observed between size at birth and size at division is incompatible with a timer mechanism. Independently of the growth law, this relation is in agreement with the incremental size control model, where the cell attempts to add a constant volume between birth and division. This incremental size control strategy was recently demonstrated in several aerobic microorganisms such as *E. coli, C. crescentus, B. subtilis*, and *S. cerevisiae* (Campos et al., 2014; Taheri-Araghi et al., 2015). We demonstrate here that the incremental model is relevant to describe the cell division of anaerobic microorganisms and would account for a universal mechanism used by aerobic and anaerobic microorganisms for cell size maintenance. The sensors responsible for this incremental strategy are still unknown but it has been suggested that replication initiation could be triggered when a critical size has been added since the last initiation event, probably through DnaA (Robert, 2015).

The localization of the Z-ring at the middle of the cells is in adequacy with the perfect symmetry of cell division observed during DvH cell cycle analysis. Fluorescent microscopic studies using an FtsZ-GFP fusion highlighted two sequential FtsZ localization patterns during the DvH cell cycle: (i), a Z-ring pattern that is clearly positioned at the midcell when the cells begin to elongate and (ii), a spot-organized pattern which is observed along the cell length in predivisional cells and in the two daughter cells after septation. This dynamic of FtsZ localization has been extensively observed in phylogenetically distant organisms (Erickson et al., 2010), suggesting a common mechanism of Z-ring assembly in DvH. However, the exact mechanism leading to the formation of the Z ring in DvH remains to be identified.

Both the formation of the Z-ring and the division site occur at the mid-cell region, suggesting that the localization of the Z-ring is also tightly regulated. Several possible mechanisms have been identified to provide strict control of symmetric/asymmetric division in model bacteria such as *E. coli, B. subtilis* or *C. crescentus*. In *E. coli* and *B. subtilis*, the spatial regulation of Z-ring positioning involves negatively acting systems that prevent Z-ring formation at the cell poles (MinCD) and over the nucleoid (SlmA in *E. coli* and Noc in *B. subtilis*) to support Z-ring formation only at the mid-cell region. MinCD is usually restricted to the poles by the topological specificity factor MinE in *E. coli* and DivIVA, which acts in concert with MinJ, in *B. subtilis* (Eswaramoorthy et al., 2011). Additional structural proteins, such as FtsA and ZipA may also play a role in stabilizing the Z-ring at the mid-cell region (Dajkovic et al., 2010). In *C. crescentus*, the spatial and temporal positioning of the Z-ring requires other alternative regulators and are supported by MipZ and ParB, which couple mid-cell localization with the initiation of chromosome replication and segregation (Thanbichler and Shapiro, 2006). It has also recently been reported in *Mycobacterium smegmatis* (Ginda et al., 2013) that ParA interacts with Wag31, a homolog of DivIVA, to mediate chromosome segregation and co-ordinate cell division. Analysis of the DvH genome reveals the presence of homologs of only some of these proteins (DivIVA, ParB, ZapA, and FtsA). It suggests that, while the stabilization mechanism is conserved, DvH have developed alternative strategies to regulate the position of cell division. Recently, we showed that proteins of the anaerobe-specific orange protein complex (ORP) share similarities with the Mrp/ParA/MinD P-loop ATPase family. A tempting hypothesis, that should be confirmed, would be that this complex would be involved in coordinating the Z-ring positioning and the chromosome segregation. In conclusion, the positioning of the Z-ring in DvH may be regulated by an undefined mechanism.

The response of DvH to oxygen has been intensively studied at both the molecular and physiological levels through transcriptomics, proteomics and functional genomics, but how oxygen interferes with cell division is unknown (Dolla et al., 2006; Zhou et al., 2011). Our data at the single-cell level show that elongation and division are differentially affected within the studied range of oxygen concentrations. From 0.02 to 0.05% oxygen, cells elongate but do not septate, ultimately leading to filamentation. These results are consistent with earlier transcriptomic studies showing that transcription of central metabolic genes are not affected during low-oxygen exposure (Mukhopadhyay et al., 2007). However, cells stop growing at higher oxygen concentrations, suggesting that both elongation and division are affected. Moreover, elongation and division are resumed when anaerobic conditions are restored, suggesting that cell division is transiently and reversibly blocked in the presence of oxygen. Bacterial filamentation primarily occurs in response to various environmental stresses as a consequence of two main mechanisms: (i) sequestration of the FtsZ protein by an interacting protein such as SulA during the SOS response (Cordell et al., 2003; Chen et al., 2012) or by inhibitors (Margalit et al., 2004; Boberek et al., 2010) or (ii) alteration of the stoichiometry of the cell-division components, such as decreased FtsZ concentration, due to either transcriptional down-regulation (Kelly et al., 1998) or FtsZ proteolysis through the action of the ClpXP protease (Camberg et al., 2009, 2011; Sass et al., 2011). The SOS response consists of approximately forty genes that are regulated by LexA and RecA, whose task is to repair DNA damage and eventually prevent cell division by the sequestration of FtsZ by SulA (Michel, 2005). Because no homolog of SulA can be identified in the DvH genome, the filamentation observed in response to oxygen may result from another mechanism. In *E. coli*, FtsZ is degraded by the two components protease ClpXP (Camberg et al., 2009, 2011). *clpX* and *clpP* genes, encoding this protease, are present in DvH genome. One can thus suppose that the mechanisms of recognition and degradation of ClpXP targets are similar in these two species. Moreover, the C-terminal residues of *E. coli* FtsZ important for ClpXP degradation of the protein (Camberg et al., 2014) are conserved in the DvH FtsZ protein. All together, these data suggest that DvH FtsZ is one of the ClpXP protease target. Further analyses are required to assess whether oxygen causes DvH filamentation by acting on the bacterial division protein FtsZ. Filamentation of anaerobic cells under oxidative conditions provided by the presence of oxygen may protect daughter cells from receiving damaged copies of the bacterial chromosome and/or eventually provide the cell sufficient time to repair oxidative damage. This transient inhibition of cell division would constitute an additional strategy that allows anaerobes to cope with the consequences of transient oxygen exposure that may be encountered in their natural environment and thus contribute to their aerotolerance.

## Author contributions

AF and AD performed the experiments and revised the draft of the manuscript. OV participated in the experiments. ARD wrote and revised the draft of the manuscript. LR, RP, and TM participated in the experiments and revised the draft of the manuscript. All the autors read and approved the final manuscript. CA designed, performed and wrote the manuscript.

### Conflict of interest statement

The authors declare that the research was conducted in the absence of any commercial or financial relationships that could be construed as a potential conflict of interest.

## References

[B1] Barák I. Wilkinson A. J. (2007). Division site recognition in *Escherichia coli* and *Bacillus subtilis*. FEMS Microbiol. Rev. 31, 311–326. 10.1111/j.1574-6976.2007.00067.x 17326815

[B2] Bernhardt T. G. de Boer P. A. J. (2005). SlmA, a nucleoid-associated, FtsZ binding protein required for blocking septal ring assembly over Chromosomes in E. coli. Mol. Cell 18, 555–564. 10.1016/j.molcel.2005.04.012 15916962 PMC4428309

[B3] Bi E. F. Lutkenhaus J. (1991). FtsZ ring structure associated with division in *Escherichia coli*. Nature 354, 161–164. 10.1038/354161a0 1944597

[B4] Boberek J. M. Stach J. Good L. (2010). Genetic evidence for inhibition of bacterial division protein FtsZ by berberine. PLoS ONE 5:e13745. 10.1371/journal.pone.0013745 21060782 PMC2966414

[B5] Boothman C. Hockin S. Holmes D. E. Gadd G. M. Lloyd J. R. (2006). Molecular analysis of a sulphate-reducing consortium used to treat metal-containing effluents. Biometals Int. J. Role Met. Ions Biol. Biochem. Med. 19, 601–609. 10.1007/s10534-006-0006-z 16946985

[B6] Camberg J. L. Hoskins J. R. Wickner S. (2009). ClpXP protease degrades the cytoskeletal protein, FtsZ, and modulates FtsZ polymer dynamics. Proc. Natl. Acad. Sci. U.S.A. 106, 10614–10619. 10.1073/pnas.0904886106 19541655 PMC2705540

[B7] Camberg J. L. Hoskins J. R. Wickner S. (2011). The interplay of ClpXP with the cell division machinery in *Escherichia coli*. J. Bacteriol. 193, 1911–1918. 10.1128/JB.01317-10 21317324 PMC3133021

[B8] Camberg J. L. Viola M. G. Rea L. Hoskins J. R. Wickner S. (2014). Location of dual sites in *E. coli* FtsZ important for degradation by ClpXP; one at the C-terminus and one in the disordered linker. PLoS ONE 9:e94964. 10.1371/journal.pone.0094964 24722340 PMC3983244

[B9] Campos M. Surovtsev I. V. Kato S. Paintdakhi A. Beltran B. Ebmeier S. E.. (2014). A constant size extension drives bacterial cell size homeostasis. Cell 159, 1433–1446. 10.1016/j.cell.2014.11.022 25480302 PMC4258233

[B10] Canfield D. E. Des Marais D. J. (1991). Aerobic sulfate reduction in microbial mats. Science 251, 1471–1473. 10.1126/science.11538266 11538266

[B11] Charvin G. Oikonomou C. Cross F. (2010). Long-term imaging in microfluidic devices. Methods Mol. Biol. 591, 229–242. 10.1007/978-1-60761-404-3_14 19957134

[B12] Chen Y. Milam S. L. Erickson H. P. (2012). SulA inhibits assembly of FtsZ by a simple sequestration mechanism. Biochemistry (Mosc.) 51, 3100–3109. 10.1021/bi201669d 22432817 PMC3518438

[B13] Cordell S. C. Robinson E. J. H. Lowe J. (2003). Crystal structure of the SOS cell division inhibitor SulA and in complex with FtsZ. Proc. Natl. Acad. Sci. U.S.A. 100, 7889–7894. 10.1073/pnas.1330742100 12808143 PMC164683

[B14] Dajkovic A. Pichoff S. Lutkenhaus J. Wirtz D. (2010). Cross-linking FtsZ polymers into coherent Z rings. Mol. Microbiol. 78, 651–668. 10.1111/j.1365-2958.2010.07352.x 20969647

[B15] de Boer P. Crossley R. Rothfield L. (1992). The essential bacterial cell-division protein FtsZ is a GTPase. Nature 359, 254–256. 10.1038/359254a0 1528268

[B16] Dolla A. Fournier M. Dermoun Z. (2006). Oxygen defense in sulfate-reducing bacteria. J. Biotechnol. 126, 87–100. 10.1016/j.jbiotec.2006.03.041 16713001

[B17] Donovan C. Schauss A. Krämer R. Bramkamp M. (2013). Chromosome segregation impacts on cell growth and division site selection in *Corynebacterium glutamicum*. PLoS ONE 8:e55078. 10.1371/journal.pone.0055078 23405112 PMC3566199

[B18] Ducret A. Maisonneuve E. Notareschi P. Grossi A. Mignot T. Dukan S. (2009). A microscope automated fluidic system to study bacterial processes in real time. PLoS ONE 4:e7282. 10.1371/journal.pone.0007282 19789641 PMC2748647

[B19] Erickson H. P. Anderson D. E. Osawa M. (2010). FtsZ in Bacterial Cytokinesis: cytoskeleton and force generator all in one. Microbiol. Mol. Biol. Rev. 74, 504–528. 10.1128/MMBR.00021-10 21119015 PMC3008173

[B20] Eswaramoorthy P. Erb M. L. Gregory J. A. Silverman J. Pogliano K. Pogliano J.. (2011). Cellular architecture mediates DivIVA ultrastructure and regulates min activity in *Bacillus subtilis*. mBio 2, e00257–e00211. 10.1128/mBio.00257-11 22108385 PMC3225972

[B21] Fiévet A. My L. Cascales E. Ansaldi M. Pauleta S. R. Moura I.. (2011). The anaerobe-specific orange protein complex of *Desulfovibrio vulgaris* hildenborough is encoded by two divergent operons coregulated by σ54 and a cognate transcriptional regulator. J. Bacteriol. 193, 3207–3219. 10.1128/JB.00044-11 21531797 PMC3133258

[B22] Ginda K. Bezulska M. Ziółkiewicz M. Dziadek J. Zakrzewska-Czerwiñska J. Jakimowicz D. (2013). ParA of *Mycobacterium smegmatis* co-ordinates chromosome segregation with the cell cycle and interacts with the polar growth determinant DivIVA. Mol. Microbiol. 87, 998–1012. 10.1111/mmi.12146 23289458

[B23] Hansen M. C. Palmer R. J. Udsen C. White D. C. Molin S. (2001). Assessment of GFP fluorescence in cells of *Streptococcus gordonii* under conditions of low pH and low oxygen concentration. Microbiol. Read. Engl. 147, 1383–1391. 10.1099/00221287-147-5-1383 11320140

[B24] Harry E. Monahan L. Thompson L. (2006). Bacterial cell division: the mechanism and its precison. Int. Rev. Cytol. 253, 27–94. 10.1016/S0074-7696(06)53002-5 17098054

[B25] Hehl A. B. Marti M. Köhler P. (2000). Stage-specific expression and targeting of cyst wall protein-green fluorescent protein chimeras in *Giardia*. Mol. Biol. Cell 11, 1789–1800. 10.1091/mbc.11.5.1789 10793152 PMC14884

[B26] Jia W. Whitehead R. N. Griffiths L. Dawson C. Bai H. Waring R. H.. (2012). Diversity and distribution of sulphate-reducing bacteria in human faeces from healthy subjects and patients with inflammatory bowel disease. FEMS Immunol. Med. Microbiol. 65, 55–68. 10.1111/j.1574-695X.2012.00935.x 22309113

[B27] Jørgensen B. B. (1982). Mineralization of organic matter in the sea bed—the role of sulphate reduction. Nature 296, 643–645. 10.1038/296643a0

[B28] Jun S. Taheri-Araghi S. (2015). Cell-size maintenance: universal strategy revealed. Trends Microbiol. 23, 4–6. 10.1016/j.tim.2014.12.001 25497321

[B29] Kelly A. J. Sackett M. J. Din N. Quardokus E. Brun Y. V. (1998). Cell cycle-dependent transcriptional and proteolytic regulation of FtsZ in *Caulobacter*. Genes Dev. 12, 880–893. 10.1101/gad.12.6.880 9512521 PMC316630

[B30] Margalit D. N. Romberg L. Mets R. B. Hebert A. M. Mitchison T. J. Kirschner M. W.. (2004). Targeting cell division: small-molecule inhibitors of FtsZ GTPase perturb cytokinetic ring assembly and induce bacterial lethality. Proc. Natl. Acad. Sci. U.S.A. 101, 11821–11826. 10.1073/pnas.0404439101 15289600 PMC511058

[B31] Michel B. (2005). After 30 years of study, the bacterial SOS response still surprises us. PLoS Biol. 3:e255. 10.1371/journal.pbio.0030255 16000023 PMC1174825

[B32] Mukhopadhyay A. Redding A. M. Joachimiak M. P. Arkin A. P. Borglin S. E. Dehal P. S.. (2007). Cell-wide responses to low-oxygen exposure in *Desulfovibrio vulgaris* Hildenborough. J. Bacteriol. 189, 5996–6010. 10.1128/JB.00368-07 17545284 PMC1952033

[B33] Mussmann M. Ishii K. Rabus R. Amann R. (2005). Diversity and vertical distribution of cultured and uncultured Deltaproteobacteria in an intertidal mud flat of the Wadden Sea. Environ. Microbiol. 7, 405–418. 10.1111/j.1462-2920.2005.00708.x 15683401

[B34] Peters P. C. Migocki M. D. Thoni C. Harry E. J. (2007). A new assembly pathway for the cytokinetic Z ring from a dynamic helical structure in vegetatively growing cells of *Bacillus subtilis*. Mol. Microbiol. 64, 487–499. 10.1111/j.1365-2958.2007.05673.x 17493130

[B35] Postgate J. R. Kent H. M. Robson R. L. Chesshyre J. A. (1984). The genomes of *Desulfovibrio gigas* and *D. vulgaris.* J. Gen. Microbiol. 130, 1597–1601. 10.1099/00221287-130-7-1597 6088669

[B36] Ravenschlag K. Sahm K. Knoblauch C. Jørgensen B. B. Amann R. (2000). Community Structure, Cellular rRNA Content, and Activity of Sulfate-Reducing Bacteria in Marine Arctic Sediments. Appl. Environ. Microbiol. 66, 3592–3602. 10.1128/AEM.66.8.3592-3602.2000 10919825 PMC92189

[B37] Robert L. (2015). Size sensors in bacteria, cell cycle control, and size control. Microb. Physiol. Metab. 6, 515. 10.3389/fmicb.2015.00515 26074903 PMC4448035

[B38] Rothfield L. Taghbalout A. Shih Y.-L. (2005). Spatial control of bacterial division-site placement. Nat. Rev. Microbiol. 3, 959–968. 10.1038/nrmicro1290 16322744

[B39] Sass H. Wieringa E. Cypionka H. Babenzien H. D. Overmann J. (1998). High genetic and physiological diversity of sulfate-reducing bacteria isolated from an oligotrophic lake sediment. Arch. Microbiol. 170, 243–251. 10.1007/s002030050639 9732438

[B40] Sass P. Josten M. Famulla K. Schiffer G. Sahl H.-G. Hamoen L.. (2011). Antibiotic acyldepsipeptides activate ClpP peptidase to degrade the cell division protein FtsZ. Proc. Natl. Acad. Sci. U. S. A. 108, 17474–17479. 10.1073/pnas.1110385108 21969594 PMC3198362

[B41] Schneider C. A. Rasband W. S. Eliceiri K. W. (2012). NIH Image to ImageJ: 25 years of image analysis. Nat. Methods 9, 671–675. 10.1038/nmeth.2089 22930834 PMC5554542

[B42] Soifer I. Robert L. Barkai N. Amir A. (2014). Single-cell analysis of growth in budding yeast and bacteria reveals a common size regulation strategy. Available online at: http://arxiv.org/abs/1410.4771 10.1016/j.cub.2015.11.067 26776734

[B43] Taheri-Araghi S. Bradde S. Sauls J. T. Hill N. S. Levin P. A. Paulsson J.. (2015). Cell-size control and homeostasis in bacteria. Curr. Biol. 25, 385–391. 10.1016/j.cub.2014.12.009 25544609 PMC4323405

[B44] Thanbichler M. Shapiro L. (2006). MipZ, a spatial regulator coordinating chromosome segregation with cell division in *Caulobacter*. Cell 126, 147–162. 10.1016/j.cell.2006.05.038 16839883

[B45] Thanedar S. Margolin W. (2004). FtsZ exhibits rapid movement and oscillation waves in helix-like patterns in *Escherichia coli*. Curr. Biol. 14, 1167–1173. 10.1016/j.cub.2004.06.048 15242613 PMC4757587

[B46] Thauer R. K. Stackebrandt E. Hamilton W. A. (2007). Energy metabolism and phylogenetic diversity of sulphate-reducing bacteria, in Sulphate-Reducing Bacteria, 1st Edn (Cambridge: Cambridge University Press), 1–38.

[B47] Treuner-Lange A. Aguiluz K. van der Does C. Gómez-Santos N. Harms A. Schumacher D.. (2013). PomZ, a ParA-like protein, regulates Z-ring formation and cell division in Myxococcus xanthus. Mol. Microbiol. 87, 235–253. 10.1111/mmi.12094 23145985

[B48] Turner J. J. Ewald J. C. Skotheim J. M. (2012). Cell size control in yeast. Curr. Biol. 22, 350–359. 10.1016/j.cub.2012.02.041 22575477 PMC3350643

[B49] Wu L. J. Errington J. (2012). Nucleoid occlusion and bacterial cell division. Nat. Rev. Microbiol. 10, 8–12. 10.1038/nrmicro2671 22020262

[B50] Zhou J. He Q. Hemme C. L. Mukhopadhyay A. Hillesland K. Zhou A.. (2011). How sulphate-reducing microorganisms cope with stress: lessons from systems biology. Nat. Rev. Microbiol. 9, 452–466. 10.1038/nrmicro2575 21572460

