# Supplementary material for: Single-Cell Analysis of Growth and Cell Division of the Anaerobe Desulfovibrio vulgaris Hildenborough
Source: Front Microbiol. 2015 Dec 8;6:1378. doi: 10.3389/fmicb.2015.01378 (PMC4672049; doi:10.3389/fmicb.2015.01378)
Supplement: Supplementary file 1 [file Table1.DOC]

Table S1. Bacterial strains and plasmids used in this study.

| Strains or plasmids | Characteristics | Reference or source |
| --- | --- | --- |
| **Strains**  *E.coli*  TG1  MW3064  *Desulfovibrio vulgaris* Hildenborough  DvH (*ftsZ-GFP*)  DvH (pBMC6 *pC_3_::gfp*)  **Plasmids**  pEGFP-N1  pNot19  pNot19Cm-Mob-XS  pNot19Cm-Mob-XS *ftsZ-gfp*  pBMC6  pBMC6 *pC_3_::gfp* | K12, Δ(*lac-pro*), *supE*, *thi*, *hsdD5* (F’, *traD36*, *proA^+^B^+^*, *lacI^Q^*, *lacZΔM15*)  Donor strain for conjugation; *thrB1004 pro thi rpsL hsdS lacZ*_*M15*  *RP4*-*1360* _(*araBAD*)*567* _*dapA1341*::_*erm pir*(*wt*)_  Wild-type strain  Contains a fusion between *ftsZ* and *gfp* genes, separated by a linker, integrated in *Dv*H genome at *ftsZ* loci.  Contains a replicative plasmid with a fusion between *cyc* gene promoter and *gfp* gene  Cloning vector, Kan^R^  Cloning vector pUC19. *Nde*I site replaced by *Not*I site  Contains the 1.4 Kb cm gene and the oriT of plasmid RP4 on a 1.6-kb fragment in the pNot19  pNot19Cm-Mob-XS contains the fusion between *ftsZ* and *gfp* separated by a linker  Cloning vector, pBG1replicon of *Desulfovibrio*, Cm^R^  Contains *pC_3_::gfp* fusion between *Hind*III and *Sac*I sites | Sambrook and Russell (2001)  Dehio et al (1997)  Postage (1984)  This study  This study  Addgene  Dolla et al (2000)  Fiévet et al (2011)  This study  Rousset et al (1998)  This study |
