# Supplementary material for: Single-Cell Analysis of Growth and Cell Division of the Anaerobe Desulfovibrio vulgaris Hildenborough
Source: Front Microbiol. 2015 Dec 8;6:1378. doi: 10.3389/fmicb.2015.01378 (PMC4672049; doi:10.3389/fmicb.2015.01378)
Supplement: Supplementary file 2 [file Table2.DOC]

**Table S2.** Primers used in this study.

| Primer | Sequence |
| --- | --- |
| Fluorescence test in DvH  NterGFP-NdeI  CterGFP-SacI  Promcyc_HindIII  Promcyc_SalI_NdeI  To construct plasmid for *ftsZlink-gfp* fusion  NterFtsZ-XhoI  CterFtsZlink-NdeI-SpeI  CterGFP-SpeI  CM1  CM2  Mob_UP  Mob_DOWN  FtsA-dir  For real-time PCR analysis  16S_left  16S_right  FtsZ_left  FtsZ_right  FtsA_left  FtsA_right  FtsQ_left  FtsQ_right  MurB_left  MurB_right  FtsI_left  FtsI_right  MraZ_left  MraZ_right | 5’-GCAGCATATGGTGAGCAAGGGCGAGGAG-3’  5’-GCAGGAGCTCTTACTTGTACAGCTCGTCCATGCCG-3’  5’-TAGCAAAGCTTCAGACAGCCCTGACATCCGGGCCT-3’  5’-TAGCAGTCGACCATATGCGAACTACCTCCTTCACAA  AG-3’  5’-CGATCTCGAGATGGAATTCATGGATATC-3’  5’-AAAATACTAGTGGCTACATATGCGGCGGCGGCGG  GTCGGCCTGCTTGCGAATGAACG-3’  5’-CGATACTAGTTTACTTGTACAGCTCGTCCAT-3’  5’-GACATGGAAGCCATCACAGA-3’  5’-ACTCCGCTAGCGCTGATGT-3’  5’-GCCGGGCGGCCGCGCGCCATTC-3’  5’-GCAGGTCGACTCTAGAGG-3’  5’-CCTGGAGCTCAAGTTCCGCATCC-3’  5’-CCTAGGGCTACACACGTACTACAA-3’  5’-GAGCATGCTGATCTCGAATTACTA-3’  5’-ATCAAGGTCATAGGCGTAGGTG-3’  5’-TGAGTTTTTCGCCAAGCTGTATC-3’  5’-TCACATCGGCTCAGAACATC-3’  5’-TGTGTGCTTGATGGAATCGT-3’  5’-AAAAGGGATCGGCAGTTTTT-3’  5’-CATCAGATTGCCCGAGATTT-3’  5’-TGCGTGCAAACTACCTGAAG-3’  5’-TGTGCCAGTTGAAGCAGTTC-3’  5’-TGTTCGGTGTCATCTGGTGT-3’  5’-GTGTTGTCCACATCCTGCAC-3’  5’-GGGTCGAGTTCGAAGACAAG-3’  5’-GGTAAGTCCGGCATAGTCCA-3’ |
