# Supplementary material for: Single-Cell Analysis of Growth and Cell Division of the Anaerobe Desulfovibrio vulgaris Hildenborough
Source: Front Microbiol. 2015 Dec 8;6:1378. doi: 10.3389/fmicb.2015.01378 (PMC4672049; doi:10.3389/fmicb.2015.01378)
Supplement: Supplementary file 3 [file Table3.DOCX]

| Part | Materials |
| --- | --- |
| Lid | - 1 x Plate of Poly(methyl methacrylate) (PMMA) [66 x 40 x 8 mm]  - 1 x O-ring sealing elastomers. [118.7 mm I.D. / 121.9 mm O.D.]  - 6 x M4 - Internal Hex Socket Cap Screws [6 mm O.D.]  - Vacuum grease |
| Adapter | - 1 x Plate of Aluminum Alloys AU 4 G [80 x 54 x 7 mm] |
| Connectors | - 2 x Tubing connectors (GE18-1003-68 Sigma, France)  - 2 x O-ring sealing elastomers. [7.16 mm I.D. / 8.16 mm O.D.]  - 2 x Type H head brass Screws. [6 mm O.D.] |

Table S3 : Material used to construct the controlled atmosphere observation chambers.
