# Supplementary material for: Single-Cell Analysis of Growth and Cell Division of the Anaerobe Desulfovibrio vulgaris Hildenborough
Source: Front Microbiol. 2015 Dec 8;6:1378. doi: 10.3389/fmicb.2015.01378 (PMC4672049; doi:10.3389/fmicb.2015.01378)
Supplement: Supplementary file 4 [file DataSheet1.DOCX]

**Figure S1. Representation of the chromosomal insertion of pNot19Cm-Mob-XS-*ftsZ-gfp* plasmid in DvH genome to form the *ftsZ-gfp* genotype.** The homologous recombination site is shown in dotted. In the scheme of DvH *ftsZ-gfp* genotype strain, all genes of the *mra* operon are not shown, only the *ftsZ* gene is annotated while other genes are regrouped under the name *mra* operon.
