# Supplementary material for: Single-Cell Analysis of Growth and Cell Division of the Anaerobe Desulfovibrio vulgaris Hildenborough
Source: Front Microbiol. 2015 Dec 8;6:1378. doi: 10.3389/fmicb.2015.01378 (PMC4672049; doi:10.3389/fmicb.2015.01378)
Supplement: Supplementary file 5 [file DataSheet2.DOCX]

**
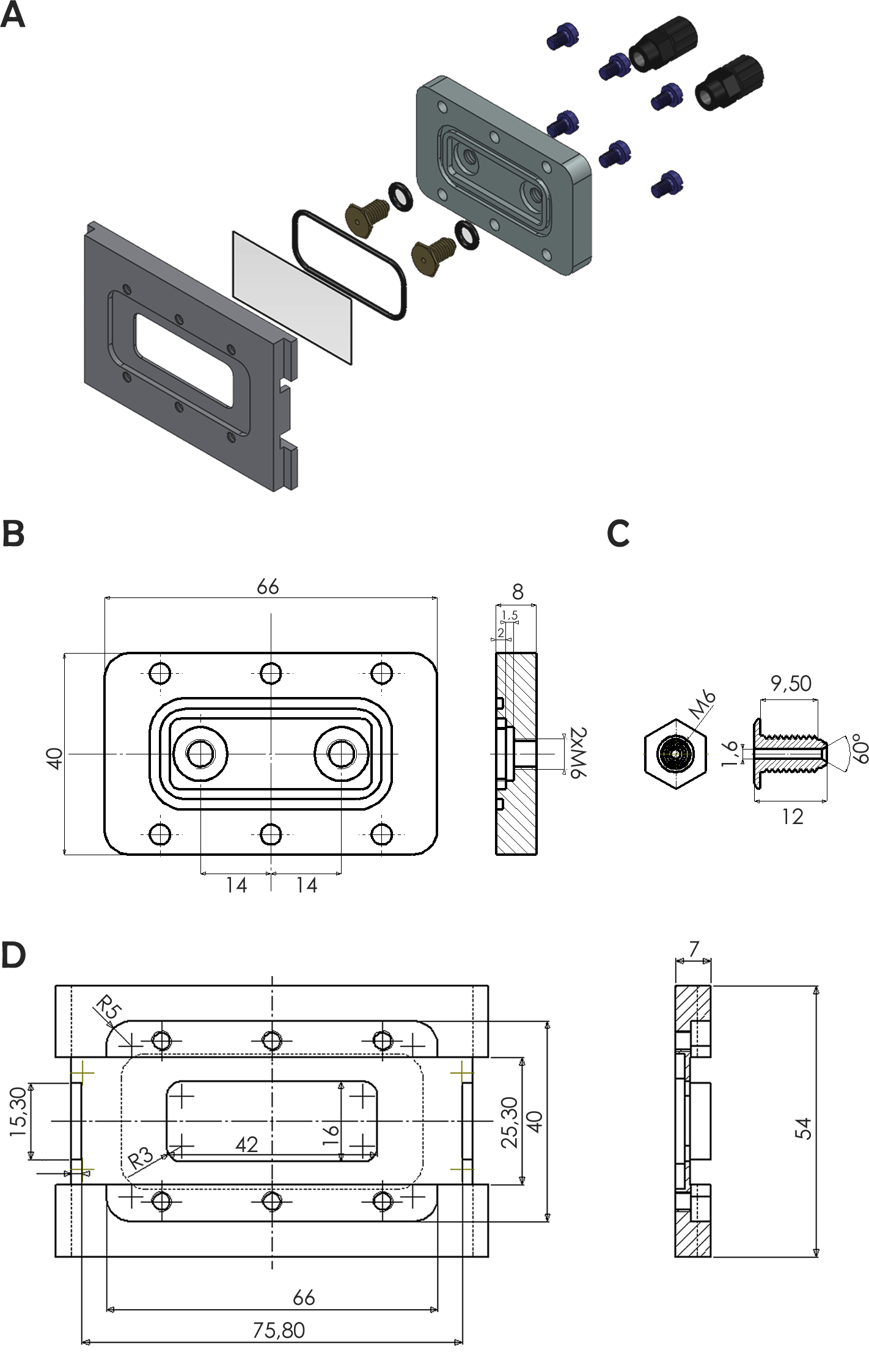
**

**Figure S2:** (A) An exploded view of the controlled-atmosphere observation chamber. (B-D) The technical drawing of the transparent lid (B), the hollowed screws (C) and the adapter (D). Dimensions are given in mm.
