# Supplementary material for: Single-Cell Analysis of Growth and Cell Division of the Anaerobe Desulfovibrio vulgaris Hildenborough
Source: Front Microbiol. 2015 Dec 8;6:1378. doi: 10.3389/fmicb.2015.01378 (PMC4672049; doi:10.3389/fmicb.2015.01378)
Supplement: Supplementary file 6 [file DataSheet3.DOCX]

**Figure S3. Absence of correlation between elongation length and birth length in DvH daughter cells.** The color of the dots (blue to yellow) represents the local density. Red dots show data binned according to the size at birth. The prediction with the incremental model is indicated with a black line.
