# Supplementary material for: Single-Cell Analysis of Growth and Cell Division of the Anaerobe Desulfovibrio vulgaris Hildenborough
Source: Front Microbiol. 2015 Dec 8;6:1378. doi: 10.3389/fmicb.2015.01378 (PMC4672049; doi:10.3389/fmicb.2015.01378)
Supplement: Supplementary file 7 [file DataSheet4.DOCX]

**D**

**C**

**A**

**B**

**F**

**E**

**Figure S4. DvH FtsZ-GFP growth parameters.** Distribution of the elongation rates (A), birth lengths (B), division lengths (C), elongation lengths (D), division time (E) and division site placement (F) of dividing DvH WT cells in black and DvH FtsZ-GFP cells in grey. (n=423 for DvH WT cells and n=380 for DvH FtsZ-GFP cells).
