# Supplementary material for: Single-Cell Analysis of Growth and Cell Division of the Anaerobe Desulfovibrio vulgaris Hildenborough
Source: Front Microbiol. 2015 Dec 8;6:1378. doi: 10.3389/fmicb.2015.01378 (PMC4672049; doi:10.3389/fmicb.2015.01378)
Supplement: Supplementary file 8 [file DataSheet5.DOCX]

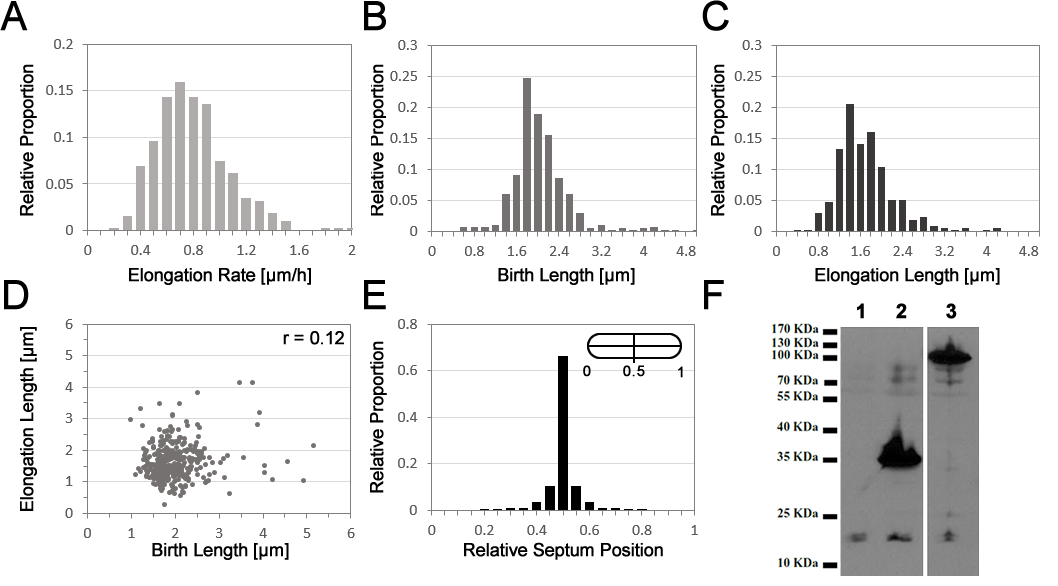


**Figure S5. Production of FtsZ-GFP fusion in DvH recombinant cells.** Western blot analysis of DvH WT cells (line 1), DvH cells producing GFP (line 2) and DvH cells producing FtsZ-GFP fusion (line 3) using an anti-GFP antibody.
