# Supplementary material for: Single-Cell Analysis of Growth and Cell Division of the Anaerobe Desulfovibrio vulgaris Hildenborough
Source: Front Microbiol. 2015 Dec 8;6:1378. doi: 10.3389/fmicb.2015.01378 (PMC4672049; doi:10.3389/fmicb.2015.01378)
Supplement: Supplementary file 9 [file DataSheet6.DOCX]

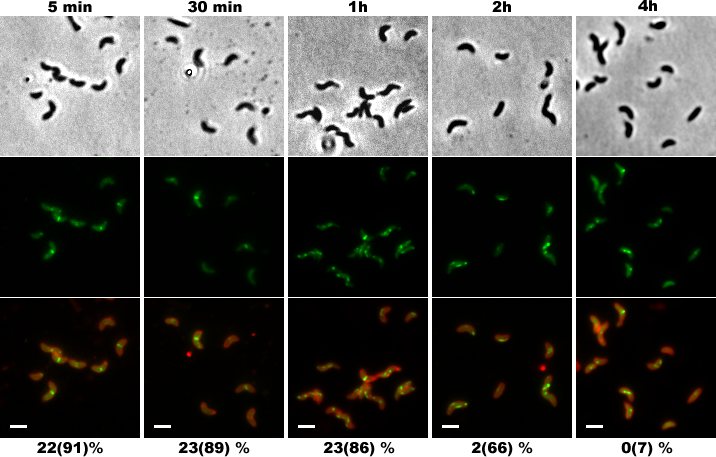


**Figure S6. Simultaneous localization of FtsZ-GFP and the cell membrane (FM4-64) after different exposure times to air.** In each case, the first row represents the phase-contrast images, the second represents the FtsZ-GFP localization and the third represents an overlay of all fluorescent signals. Scale bar = 1 µm. Each percentage represents the cells exhibiting FtsZ-GFP as a Z-ring structure. The percentage in brackets represents cells with a single focus of FtsZ-GFP localized along the cells or as a Z-ring.
